# Oxidative Stress-Induced Protein of SESTRIN2 in Cardioprotection Effect

**DOI:** 10.1155/2022/7439878

**Published:** 2022-07-29

**Authors:** Huang Rongjin, Chen Feng, Ke Jun, Lin Shirong

**Affiliations:** Shengli Clinical Medical College of Fujian Medical University/Department of Emergency, Fujian Provincial Hospital/Fujian Key Laboratory of Emergency Medicine, Fujian Provincial Hospital/Fujian Provincial Institute of Emergency Medicine/Fujian Emergency Medical Center, Fuzhou 350001, China

## Abstract

Because of the rich mitochondria and high energy metabolic requirements, excessive oxidative stress generated by ROS is a key pathogenic mechanism in heart disease. SESTRIN2, the well-known antioxidant protein, plays a vital role in diminishing the production and accumulation of ROS, thus sparing cells from oxidative damage. From this new perspective, we first examine SESTRIN2 structure-function relationships; then, we describe how SESTRIN2 expression is regulated under oxidative stress conditions, emphasizing SESTRIN2's antioxidant mechanism via multiple signal transductions; and finally, we discuss SESTRIN2's role in a variety of oxidative stress-related cardiac diseases, including age-related heart disease, diabetic cardiomyopathy, ischemia-reperfusion myocardial injury, septic cardiomyopathy, and chronic cardiac insufficiency. The goal of this review is to identify the SESTRIN2 protein as a potential biomarker and new therapy target for oxidative stress-related cardiac diseases.

## 1. Introduction

The 2020 updated statistics of AHA reported that the prevalence of cardiac diseases is still increasing and one of the death threats worldwide, contributing to increased morbidity, mortality, and healthcare costs. In particular, with an aging and rapid increasing in the size of the elderly population in China, there is an urgent need to develop interventions aimed at preventing cardiac disease [1]. To optimize interventions and improve clinical outcomes, we need to search for novel diagnostic markers and therapeutic targets for cardiac diseases. Currently, accumulating evidence has shown that oxidative stress plays a crucial role in cardiac diseases [2, 3]. Therefore, targeting of ROS signaling pathways may be a promising strategy for cardiac diseases prevention and therapy [4]. SESTRIN2, an oxidative stress-inducible protein, may function as the principal signaling molecule in this system, because convincing evidence suggests that SESTRIN2 plays a pivotal role in maintaining normal heart physiology [5], and the outcome of several subsequent study with respect to SESTRIN2 may indicate its potential protective role in cardiac diseases due to various stress states [6]. Accordingly, we first summarize the biological characteristics of SESTRIN2 and describe the regulatory mechanisms of SESTRIN2 under oxidative stress. Next, we elucidate the pathophysiological effects of SESTRIN2 signal transduction against oxidative stress. Finally, we emphasize the current research progresses on SESTRIN2 and oxidative stress-related cardiac diseases, aiming to sort out SESTRIN2 as a potential biomarker and therapeutic target for cardiac diseases based on the new perspective of oxidative stress-inducible SESTRIN2 expression and cardiac function protection.

## 2. Structural and Functional Basis of SESTRIN2 (Figure 1)

### 2.1. Structure of SESTRIN2 Protein

Mammalian SESTRIN proteins comprise SESTRIN1, SESTRIN2, and SESTIRIN3, which share around 50% of their amino acid sequence. These isoforms of SESTRINs, respectively, expressed by three specific coding genes *SESN1*, *SESN2*, and *SESN3*. The *hSESN1* gene, located at chromosome 6p21, is a member of the GADD gene family, also known as PA26, which is triggered by serum deprivation and growth arrest [7]. The *hSESN2* gene, which is found on chromosome 1p35.3, increases transcription in response to persistent hypoxia and was previously known as Hi95 [8]. The *hSESN3* gene, which is found on chromosome 11q21 and can be controlled by the FOXO subfamily of nuclear transcription factors [9], has received less attention. The research of *SESN2* gene expression, protein structure, and functional features is now gaining popularity. Budanov et al. found the *SESN2* gene in 2002 after studying the ORF of the nucleotide sequence of the cDNA microarray of the human glioma cell line A172 after a prolonged duration of hypoxia induction [8]. SESTRIN2 mRNA may be quantified in a range of normal human tissues using next-generation sequencing [10], with the potential to encode 480 amino acids and translate to a protein of around 55-60 kDa in size. Its expression was found in the cytoplasm [9], while a subsequent investigation found it in the mitochondria [11]. Several transcription factors, including Nrf2 and HIF-1a, are involved in its control [12, 13]. The recently identified crystal structure of the human SESTRIN2 protein shows that the protein contains an N-terminal globular domain (*SESN*-A) and a C-terminal globular domain (*SESN*-C) linked by a helix-loop-helix domain (*SESN*-B) [14]. The *SESN*-A domain contains the catalytic cysteine (C125), which acts as an active site for inhibiting ROS activity, as well as conserved proton relay system residues (Try127 and His132), which bind to SQSTM1/p62 and ULK1 [14, 15]. The *SESN*-C structural domain [14, 16], which contains a distinctive aspartate-aspartate (Asp406 and Asp407, DD) motif, interacts with GATOR2, blocking the mTOR signaling pathway. In addition, the *SESN*-C domain has a leucine-binding site that forms large van der Waals contacts with Leu389, which has been discovered to be critical for regulating mTOR expression by accepting variations in leucine signaling [17, 18].

### 2.2. Functional Basis of SESTRIN2 Protein

Functional studies show that SESTRIN2 protein may participate in biological processes such as gene transcription, protein translation, and cellular autophagy in a variety of normal human tissues, implying that it can help maintain tissue and organ function. In numerous animal models, genetic depletion of *Sesn2* has accelerated the evolution of a variety of age- and obesity-related pathological illnesses, such as fat accumulation, muscle degeneration, insulin resistance, cardiac dysfunction, mitochondrial pathologies, and carcinogenesis [10, 16, 19]. SESTRIN2 is a conserved stress-inducible protein that is activated by a variety of noxious stimuli such as DNA damage, hypoxia, oxidative stress, and metabolic stress [16]. Upregulated SESTRIN2 expression has a cytoprotective effect by influencing pathological states like oxidative stress [16], autophagy [20], and endoplasmic reticulum stress [21], causing changes in relevant signaling pathways that protect against diseases like cardiovascular disease, metabolic disease, neurodegenerative disease, and cancer. SESTRIN2 protein loss or dysregulation, on the other hand, can hasten illness development [22].

## 3. Expression of SESTRIN2 Protein under Oxidative Stress (Figure 2)

### 3.1. Various Transcription Pathways of *SESN2* Induction under Oxidative Stress

The energy required for life is provided by the biological oxidation of nutrients. During oxidative phosphorylation, electrons leaking from mitochondrial complexes in the oxidative respiratory chain, also known as the electron transport chain, generates ROS such as oxygen radical superoxide anion (O2-) and hydroxyl radical (OH-) and nonoxygen radical hydrogen peroxide (H2O2) [23]. The generation of ROS in a functional mitochondrion is balanced by a strong antioxidant defense capability for scavenging ROS. Mn-SOD, CAT, GPx antioxidants, and Trx-centered sulfhydryl redox system are the most significant. Mn-SOD catalyzes superoxide radical breakdown into molecular oxygen (O2) and H2O2, whereas CAT and GPx convert H2O2 into H2O and O2 [24]. A redox imbalance in favor of excess ROS leads to so-called OS. Upon oxidative stress, the expression of SESTRIN2 protein is regulated mainly by the P53 and Nrf2 but also by C/EBP*β*, FOXO3, AP-1, and NF-*κ*B [25].

### 3.2. Regulation of SESTRIN2 Protein Expression by P53 and Nrf2

Although Budanov et al. [8, 26] discovered that P53 could induce SESTRIN2 protein expression under genotoxic stress conditions, Ishihara et al. [27] found that exposing the rat kidney cell NRK-52E cell line to oxidative stress conditions produced by exogenous H2O2 upregulated SESTRIN2 protein expression in a P53-dependent manner in vitro experiments. In response to oxidative stress, P53 can regulate the expression of SESTRIN2 to avoid further cellular damage [28, 29]. In addition, using luciferase analysis, Deng et al. [29] discovered a conserved p53 regulatory region in the first intron of *SESN2/Sesn2* in both mice and humans, suggesting that p53 directly regulates SESTRIN2 expression. In contrast to P53, the regulation of SESTRIN2 protein expression by the transcription factor Nrf2 is currently being examined more thoroughly. Using hepatocellular carcinoma cell lines, Shin et al. [30] confirmed for the first time that activation of Nrf2 increased the levels of SESTRIN2-mRNA and SESTRIN2-protein. Glucose deprivation experimental conditions that were directly investigated yielded a comparable result [31]. And when Nrf2 was eliminated, the ability of Nrf2 activators to induce SESTRIN2 was completely lost [30]. Recently, hemin dose-dependently increased SESTRIN2 expression under an oxidative stress through an Nrf2-dependent mechanism, according to Kim et al. [32], following p53 mutation in mice colon tumors. Furthermore, the ARE of the *SESN2/Sesn2* gene promoter [550-539 bp sequence of human *SESN2* gene and roughly 657-646 bp sequence of mouse *Sesn2* gene] has been identified as a region of direct interaction with Nrf2 [30].

## 4. Antioxidant Mechanism of SESTRIN2 (Figure 2)

### 4.1. Direct Inhibition of ROS

New insights into human SESTRIN2's direct interaction with ROS were not achieved until Kim et al. [14] employed X-ray crystallography to establish the structure of the protein. The catalytic cysteine (Cys125) in the *SESN*-A domain works as an active site for suppressing ROS activity. SESTRIN2 has only one conserved active cysteine residue (Cys125), which is only reductive for isopropylbenzene hydroperoxides in vitro, but isopropylbenzene hydroperoxides are not present in the type of ROS produced in human tissue cells, so its specific substrate for the pathophysiological state of ROS in vivo needs to be determined further. Hydrophobic ROS might be a potential substrate based on the *SESN*-A structural domain binding ROS active site surrounded by hydrophobic surface residues [14]. This challenge is expected to be overcome with the continued development of tools for determining particular kinds of ROS.

### 4.2. Inhibition of ROS Generation

In mammalian cells, NADPH oxidase is a major generator of ROS. SESTRIN2's antioxidative stress action is also linked to the suppression of NADPH oxidase and consequently ROS generation. According to the findings of Yang et al., the mRNA levels of NADPH oxidase components (gp91phox, p47phox, and p22phox) were markedly decreasing in RAW264.7 cells stably expressing *SESN2*. These results indicate that SESTRIN2 has a cytoprotective impact against LPS-induced ROS generation and cell death through inhibition of NOX in macrophages [33]. Recently, Hwang et al. [34] used SESTRIN2 knockout mice to create embryonic fibroblasts with a ROS-related senescence phenotype and discovered that the loss of SESTRIN2's inhibitory action mediated an increase in ROS generation caused by NOX4, a member of the NADPH oxidase family. Their findings also revealed that the loss of SESTRIN2 promoted cellular senescence via ROS generation but not antioxidant protein levels.

### 4.3. Induction of Antioxidant Enzymes

1. The early finding of the *Sesn2* gene suggested that its activation and expression had antioxidant biological effects based on the structural similarity of its N-terminal structural domain to that of the AhpD protein of Mycobacterium tuberculosis [35]. AhpD is one of the enzymes involved in the degradation of AhpC, a bacterial Prx. However, SESTRIN2 only has one conserved cysteine with redox activity, Cys125, whereas AhpD has two disulfide-linked cysteines that are required for redox activity [36]. The fact that pure SESTRIN2 lacked cysteine sulfite reductase activity was later verified [37]. Further mechanistic investigations by Budanov et al. demonstrated that its upregulation of peroxisomal function may be linked to the TrxR system [36]. Under highly oxidizing conditions, Prxs lose peroxidase activity due to the over oxidation of cysteine to sulfinic acid (Cys-SO2H) or sulfonic acid (Cys-SO3H) in the active site. And sufinylated Prxs are reactivated by Srx and Trx [38]. The key to SESTRIN2's antioxidative stress mechanism, then, may be in the indirect control of Prx and other peroxidases expression
2. The facilitation of nuclear translocation of Nrf2, which contains a highly conserved bZIP structure, based on which six structural and functional domains are distinguished and named Neh1-Neh6(Nrf2-ECH homology), is currently thought to be the most important bridging node in the indirect regulation of Prx and other peroxidases expression by SESTRIN2. Nrf2 is found in the cytoplasm under normal circumstances and binds to the Kelch domain of KEAP1 via the N-terminal Neh2 domain. KEAP1 is a cysteine-rich protein with a BTB domain that binds to Cul3 to compose Keap1-Cul3-Rbx1 complex, promoting the ubiquitination and degradation of Nrf2 protein to maintain a low level in nonstress state [39].
3. During oxidative stress, ULK1 and SQSTM1/p62, but not the regulatory subunits Atg13 and FIP200 of ULK1, were found to physically interact with SESTRIN2, thus promotes phosphorylation of SQSTM1/p62. SQSTM1/p62 is activated, and it competes with Nrf2 for KEAP1 binding while also attracting the autophagy-associated protein to create the LC3-p62-Keap1 ternary complex, which causes lysosomal degradation of Nrf2 activity. As a result, Nrf2 tranlocates into the nucleus, where it binds to a specific DNA sequence known as the ARE, and regulates the expression of cellular antioxidant genes like SESTRIN2, Srx, Trx, and Prx (all of which have ARE structures) to protect cells from oxidative stress damage [15, 40]. Besides, as mentioned above, SESTRIN2 is a direct transcriptional target of Nrf2, resulting in a positive feedback loop in the SESTRIN2-Keap1-Nrf2 signaling axis, maximizing the activity of Nrf2-ARE activation in eliminating ROS accumulation. This has been confirmed by a large number of subsequent researches

### 4.4. Upregulation Mitochondrial Autophagy

The accumulation of damaged mitochondria also leads to oxidative stress, which results in elevated levels of ROS. SESTRIN2 has been found to upregulate mitochondrial autophagy to remove damaged mitochondria and ROS in both AMPK-dependent and non-AMPK-dependent ways. Dependent on AMPK phosphorylation and subsequent activation of ULK1 [41], SESTRIN2 stimulates AMPK, which activates TSC2 to create the TSC1/2 complex, which removes Rheb GTP form (Rheb-GTP) to GDP form (Rheb-GDP), therefore adversely inhibiting the mTORC1 signaling pathway to upregulate mitochondrial autophagy [42]. The actual location of interaction between the SESTRIN2 structural domain and AMPK, on the other hand, is unclear. The crystal structure of the SESTRIN2 protein was also discovered to be nondependent on AMPK activity [14]. SESTRIN2 controls mTORC1 signaling in AMPK-knockout mice cells by acting as guanine nucleotide dissociation inhibitors for Rag GTPases. SESTRIN2 interacts with GATOR2 to release GATOR2/GATOR1 from the complex, inhibiting RagA/B activity and thereby adversely affecting the mTORC1 signaling pathway for mitochondrial autophagy upregulation [43, 44]. The *SESN*-C structural domain [14] features a unique aspartate-aspartate (Asp406 and Asp407, DD) motif, which is where they physically engage. Recently, SESTRIN2 has also been shown to enhance mitophagy via ULK1-mediated Beclin1 phosphorylation [45]. Even more fascinating is new research demonstrating that ULK1 directly phosphorylates Ser-73 and Ser-254 residues of the *SESN*-A structural domain. These findings highlight the ULK1-SESTRIN2 pathway as an optimal route for inducing mitophagy quickly [46].

## 5. SESTRIN2 and Oxidative Stress-Related Cardiac Diseases (Table 1)

### 5.1. Age-Related Heart Disease

The buildup of oxidative stress products causes aging, and many cardiac diseases, such as age-related heart disease, are linked to ageing [47]. In Drosophila, Lee et al.'s colleagues found that deleting *Sesn2* causes age-associated pathologies including cardiac malfunction [5]. They also discovered that *dSesn* knockout mutants exhibit an accelerated aging phenotype [48]. Ren et al. [49] recently demonstrated that *Sesn2* mutant animals had an aged phenotype, as well as a disordered myocardium, when exposed to high levels of oxidative stress. SESTRIN2 stimulates AMPK, inhibits mTORC1, promotes autophagy, and decreases ROS generation from defective mitochondria, reducing the aging process, according to the researchers. Furthermore, it is well known that growing older increases myocardial vulnerability to ischemia/reperfusion damage [50], which was confirmed by Lee et al., who showed that *Sesn2* mutant mice exhibit transcriptome changes comparable to those of aged mice in response to I/R stress [48]. The processes differ from the aforementioned in that they promote mitochondrial regeneration. The AMPK/peroxisome PGC-1*α* signaling pathway sensitivity to ischemia-reperfusion cardiac damage is reduced when *Sesn2* is delivered by an adeno-associated virus [51]. What is more, Ren et al. colleagues [52] used transcriptomic, proteomic, and metabolomic investigations to indicate that SESTRIN2 is important for sustaining mitochondrial function. In terms of clinical evidence, a small pilot investigation indicated that blood SESTRIN2 levels were considerably lower in 51 old and frail patients compared to 41 nonelderly controls [53], suggesting that serum SESTRIN2 levels decline with frailty in the elderly.

### 5.2. Diabetic Cardiomyopathy

There is no universal agreement on the diagnostic procedures and therapies for DCM due to the definition of diabetes. However, research on diabetic cardiomyopathy has identified a distinct physiopathogenesis. The role of oxidative stress in the development of diabetic cardiomyopathy is becoming more widely recognized [54]. Through the use of a diabetic cardiac I/R rat model, Zhou et al. [55] discovered that SESTRIN2 may boost antioxidant effects and reduce diabetes cardiac I/R mitochondrial oxidative stress damage by interacting with Nrf2. However, other academics have a different viewpoint. Zhang et al. [56] recently set out to investigate the role of SESTRIN2 in diabetic cardiomyopathy using H9C2 cardiomyocytes and DCM-induced C57BL/6 mice. Their findings suggest that inhibiting SESTRIN2 expression improves cardiac dysfunction in DCM, possibly due to the restoration of mitochondrial dysfunction-induced apoptosis. Chung et al. [57] initially discovered a tendency for elevated serum SESTRIN2 levels in people with type 2 diabetes in clinical investigations, using a large cross-sectional survey research. Clinical investigations, on the other hand, have shown mixed outcomes. Sundararajan et al. [58] show that mRNA expressions of *SESN2* are considerably lower in type 2 diabetes in a case-control study. This might be due to changes in the control of mRNA to protein translation.

### 5.3. Ischemia-Reperfusion Myocardial Injury

Reactive oxygen species production can spike during myocardial ischemia-reperfusion [59]. Liu et al. [60] demonstrated that SESTRIN2 can act as an endogenous antioxidant to protect the mouse heart from I/R injury by reducing ROS production, as evidenced by adeno-associated virus delivery of *Sesn2* into a *Sesn2*-knockout mice model and activation of ROS-related signaling pathway molecules in the disease model, as well as improved cardiac function. Morrison et al. previously found that SESTRIN2 protects against cardiac I/R injury by interacting with LKB1 to activate AMPK and increase mitochondrial autophagy, which clears damaged mitochondria and ROS [61]. Furthermore, Liu et al. [60] successfully proved that SESTRIN2, as an endogenous antioxidant, can maintain intracellular redox homeostasis under ischemia stress using a combined animal gene deletion experiment and in vitro cell culture assay. Clinical studies have reported higher plasma SESTRIN2 levels in 44 patients with stable coronary artery disease than in 35 patients without coronary artery disease [62], and preliminary findings from a recent cross-sectional study by Professor Kishimoto and his team also showed that plasma SESTRIN2 levels were higher in patients with coronary artery disease and correlated with the severity of coronary artery disease compared with noncoronary artery disease patients [63]. These clinical findings suggest that plasma SESTRIN2 may be a compensatory response to increased oxidative stress and aims to prevent the progression of coronary artery disease.

### 5.4. Septic Cardiomyopathy

Despite the lack of a universally accepted set of diagnostic criteria, there is a growing clinical tendency to refer to septic cardiac dysfunction as SICM [64]. Cardiomyopathy is defined as a condition in which the mechanical and/or electrical activity of the heart is abnormal in the absence of coronary artery disease, hypertension, heart valve disease, or congenital heart disease to explain these abnormalities, which are most commonly manifested as inappropriate hypertrophy or dilatation of the ventricles [65]. The mechanism of impaired energy metabolism due to myocardial mitochondrial oxidative stress plays a crucial role in the pathophysiology of septic cardiomyopathy [24], in which oxidative stress-induced SESTRIN2 may be a key molecule. Hwang et al. [66] showed that sesn2 knockdown reduced the AMPK phosphorylation in an in vitro model of the cardiac H9C2 cell line and an in vivo model in C57BL/6 mice, resulting in downregulation of antioxidant enzyme expression including peroxidase and superoxide dismutase, leading to increased ROS production in LPS-treated models of septic cardiomyopathy and increased expression of LPS-mediated myocardial fibrosis factor such as type I and type III collagen, resulting in impaired cardiac function. We observed that a prior clinical observational study by Kim's research team demonstrated increased levels of SESTRIN2 protein expression in blood mononuclear cells in eight patients with septic shock compared to healthy controls [67]. However, the clinical sample size was modest, and no changes in SESTRIN2 in septic cardiomyopathy serum were monitored following recovery from septic shock or an aggravation of the condition like septic cardiomyopathy. The link between serum SESTRIN2 protein expression and septic cardiomyopathy, however, is unknown.

### 5.5. Chronic Cardiac Insufficiency

The development of cardiac insufficiency at the end stage of multiple circulatory diseases, including the oxidative stress-related cardiac diseases described above, is a common outcome, with ventricular remodeling, including cardiomyocyte hypertrophy and altered cardiomyocyte phenotype, being a very important compensatory modality, with oxidative stress being involved in the pathophysiology of cardiac hypertrophy [68]. Hypertrophy of NRCM caused by oxidative stress in the model group, Du et al.'s colleagues [69] discovered that Keap1 expression was elevated, SESTRIN2 expression was downregulated, and Nrf2 and HO-1 expression were likewise downregulated in myocardial tissue. The findings imply that altering SESTRIN2 expression in the Keap1/Nrf2/HO-1 signaling pathway may be an important target for avoiding myocardial remodeling. Zhang et al. [70] used conditional deletion of AMPK2 to show that the functions and processes of SESTRIN2 overexpression in preventing pressure overload-induced cardiac hypertrophy may be dependent on the AMPK2 pathway. In clinical studies, Wang et al. [71] measured significantly elevated plasma SESTRIN2 levels in 220 patients with heart failure and found that its concentration was positively correlated with NT-pro-BNP, negatively correlated with LVEF, positively correlated with major adverse cardiac events, and progressively increased with increasing severity of cardiac function class. High plasma levels of SESTRIN2 in patients may signify a compensatory response to heart failure and may assist to avoid unfavorable cardiac events, according to the findings.

## 6. Conclusion and Prospects

The above data that are presented from multiple experimental systems, including in vitro experiments and in vivo rodent and human studies, indicate that SESTRIN2 is a promising target for the treatment of oxidative stress-related heart disease in humans. In this review, we have also briefly discussed the upstream inductive factors that modulate SESTRIN2 under oxidative stress condition and downstream signaling pathways, suggesting that SESTRIN2 quenches oxidative stress injury via activating genes expression of downstream genes of the Nrf2 pathway and the AMPK/mTOR pathway. Although preclinical models have contributed to the understanding of SESTRIN2 in cardio-protective mechanisms, to what extent these findings are applicable to humans remains unclear. Currently, human studies have only focused on the relationship between SESTRIN2 and clinical biochemical parameters and prognosis, and there is a lack of study on the main source and role of high serum SESTRIN2 levels in patients with cardiac diseases. Therefore, more clinical trials are necessary to clarify the exact mechanisms of SESTRIN2 in cytoprotection against oxidative stress conditions, providing a wide platform for the future development of novel therapeutic targets for oxidative stress-related heart disease.

## Figures and Tables

**Figure 1:**
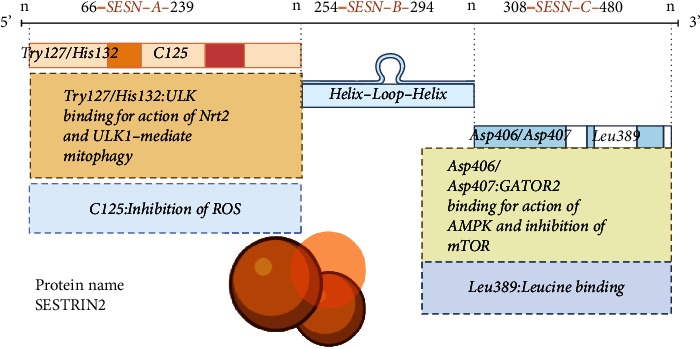
Schematic representation of the full-length gene sequence of *SESN2* showing the three structural domains *SESN*-A, *SESN*-B, and *SESN*-C; schematic representation of the spatial structure of SESTRIN2 showing the *SESN*-A redox site (C125), the *SESN*-C physical interaction site with GATOR2 and the leucine binding site.

**Figure 2:**
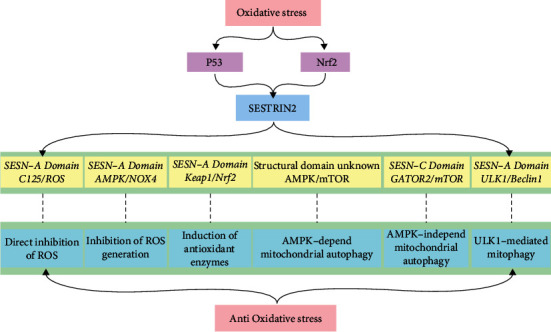
Schematic diagram of the biological effects of SESTRIN2 against oxidative stress.

**Table 1:** Summary of research progress on SESTRIN2 and oxidative stress-related heart disease.

In vitro and in vivo cellular assays			SESTRIN2			Clinical study information		
Function	Mechanism	Model	Oxidative stress-related heart disease			Subjects	Specimens	Results
AMPK-dependent mitochondrial autophagy	AMPK/mTORC	C57BL/6 mice	[49]	Age-related heart disease	[53]	51 elderly and frail individuals than 41 nonelderly controls	Serum	Aged frail less than nonaged frail
Upregulated antioxidant enzyme	Nrf2	SD rats	[55]	Diabetic cardiomyopathy	[53]	46 without diabetes and 194 with diabetes	Serum	Increasing level in subject with type2 diabetes
AMPK-independent mitochondrial autophagy	LKB1/AMPK	C57BL/6 mice	[61]	I/R myocardial injury	[62]	175 coronary patients vs. 129 noncoronary patients	Plasma	Coronary heart disease higher than noncoronary patients
Upregulated antioxidant enzyme	Keap1/Nrf2	Wistar rats	[55]		[63]	114 patients with CAD (44 SA, 41 UA and 29 AMI) and 35 patients without CAD	Plasma	CAD was higher than that of non-CAD patients, and UA and AMI were higher than SA
Upregulated antioxidant enzyme	AMPK kinase	H9C2 cell line and C57BL/6 mice	[64]	Septic shock/septic cardiomyopathy	[67]	8 patients with septic shock	Blood mononuclear cells	Higher in septic shock than in patients without septic shock
Upregulation of antioxidant enzymes to prevent myocardial remodeling	Keap1/Nrf2/HO-1	Neonatal rat cardiomyocytes	[69]	Cardiac insufficiency	[71]	20 patients with and 80 patients without heart failure	Plasma	Heart failure patients over patients without heart failure

## Abbreviations

AHA: American Heart Association
ROS: Reactive oxygen species
hSESN: Human SESN
GADD: Growth arrest and DNA damage
PA26: p53 activator gene 26
Hi95: Hypoxia-inducible gene 95
FOXO: Forkhead box protein O
ORF: Open reading frame
Nrf2: Nuclear factor-E2-related factor2
HIF-1a: Hypoxia-inducible factor-1a
SQSTM1/p62: Sequestosome-1
ULK1: UNC-51-like protein kinase 1
GTPase: Guanosine triphosphatase
Rag2: Recombination activating genes 2
GATOR2: GTPase activating protein activity toward Rag 2
mTOR: Mammalian target of rapamycin
Mn-SOD: Manganese superoxide dismutase
CAT: Catalase
GPx: Glutathione peroxidase
Trx: Thioredoxin
OS: Oxidative stress
C/EBP*β*: CCAAT-enhancer-binding protein beta
FOXO3: Forkhead box O3
AP-1: Activator protein-1
NF-*Κ*b: Nuclear factor kappa-B
ARE: Antioxidant response element
NADPH: Nicotinamide adenine dinucleotide phosphate
NOX4: NADPH oxidase 4
AhpD: Alkyl hydroperoxidase
Prxs: Peroxiredoxins
TrxR: Thioredoxin reductase
Srx: Sulfiredoxin
bZIP: Basic region-leucine zipper
KEAP1: Kelch-like ECH-associated protein 1
LC3: Microtubule-associated protein 1 light chain 3
Cul3: Cullin3
Rbx1: Ring box proein-1
dSesn: Drosophila Sesn
PGC-1*α*: Proliferator-activated receptor gamma coactivator-1*α*
DCM: Diabetic cardiomyopathy
LKB1: Liver kinase B1
SICM: Sepsis induced cardiomyopathy
NRCM: Neonatal rat cardiomyocytes
NT-pro-BNP: N-terminal B-type natriuretic peptide
LVEF: Left ventricular ejection fraction
HO-1: Heme oxygenase-1
SA: Stable angina
UA: Unstable angina
AMI: Acute myocardial infarction
AMPK: Adenosine monophosphate-activated protein kinase
Asp407-Asp406: Asp motif
CAD: Coronary artery disease.
